# T7 RNA Polymerase Functions *In Vitro* without Clustering

**DOI:** 10.1371/journal.pone.0040207

**Published:** 2012-07-02

**Authors:** Kieran Finan, Joseph P. Torella, Achillefs N. Kapanidis, Peter R. Cook

**Affiliations:** 1 Sir William Dunn School of Pathology, University of Oxford, Oxford, United Kingdom; 2 Clarendon Laboratory, Department of Physics, University of Oxford, Oxford, United Kingdom; German Cancer Research Center, Germany

## Abstract

Many nucleic acid polymerases function in clusters known as factories. We investigate whether the RNA polymerase (RNAP) of phage T7 also clusters when active. Using ‘pulldowns’ and fluorescence correlation spectroscopy we find that elongation complexes do not interact *in vitro* with a *K_d_*<1 µM. Chromosome conformation capture also reveals that genes located 100 kb apart on the *E. coli* chromosome do not associate more frequently when transcribed by T7 RNAP. We conclude that if clustering does occur *in vivo*, it must be driven by weak interactions, or mediated by a phage-encoded protein.

## Introduction

Mounting evidence suggests that many RNA and DNA polymerases function in clusters rather than in isolation. Mammalian RNA polymerase II (RNAP II), for example, appears to be active in ‘factories’ which typically contain ∼8 enzymes working on different templates, and DNA polymerases cluster in analogous ‘replication factories’ [1], [2], [3]. Such ‘factories’ may also exist in some [4], [5], [6] – but perhaps not all [7] – bacteria.

The single-subunit RNA-dependent RNA polymerases of many human viruses also cluster, forming large membrane-bound arrays in which individual molecules interact directly [8], [9]. The formation of these assemblies can have strong effects on RNAP function; poliovirus RNA-dependant RNAPs, for example, cannot transcribe efficiently without forming clusters [10].

Although there are many ways in which the cell might benefit from the existence of polymerase clusters [1], the evolutionary forces responsible for their formation remain poorly understood. One possibility is that clustering creates a high local concentration that facilitates nucleic acid synthesis [11]. Another is that RNAP clustering evolved because freely-mobile enzymes would track along and rotate about their templates, and so entangle their trailing nascent transcripts; conversely, RNAPs immobilized in clusters would reel in their templates without rotating, and so extrude unentangled transcripts [11].

The RNAP of bacteriophage T7 is one of the best studied DNA-dependant RNAPs. The conformation of this single-subunit enzyme remains largely unchanged during promoter binding and polymerization of the first three nucleotides [12], [13], [14]; however, by +7, the enzyme has already undergone significant rearrangements [15] and by +14 has morphed into its final processive form [16], [17]. The resulting elongation complex (EC) is highly stable [18], and transcribes at ∼50–200 bp/s [19], [20].

Little is known about the clustering of any of these T7 RNAP isoforms. However the unengaged enzyme does ‘aggregate’ at the high concentrations (∼10 µM) used during purification and crystallization [21], [22], [23] – and so is often solubilized using non-physiological concentrations of NaCl and glycerol [24], [25]. It is not known whether this interaction is physiologically relevant, or occurs at lower RNAP concentrations.

Whether ECs cluster is equally unclear. Although isolated monomers can function when immobilized *in vitro* [19], [26], it remains to be seen whether ECs cluster *in vivo* or in solution. ECs have been imaged by atomic force microscopy and appear as monomers [27]; however, the procedures used to prepare these samples may have destroyed any pre-existing clusters.

Here, we investigate whether or not T7 RNAP ECs cluster using ‘pulldowns’, fluorescence correlation spectroscopy, and chromosome conformation capture. We find no evidence for clustering, and conclude that if it does occur *in vivo*, it is probably driven by weak interactions.

## Results

### T7 RNAP ECs do not co-associate *in vitro*

**Figure 1:**
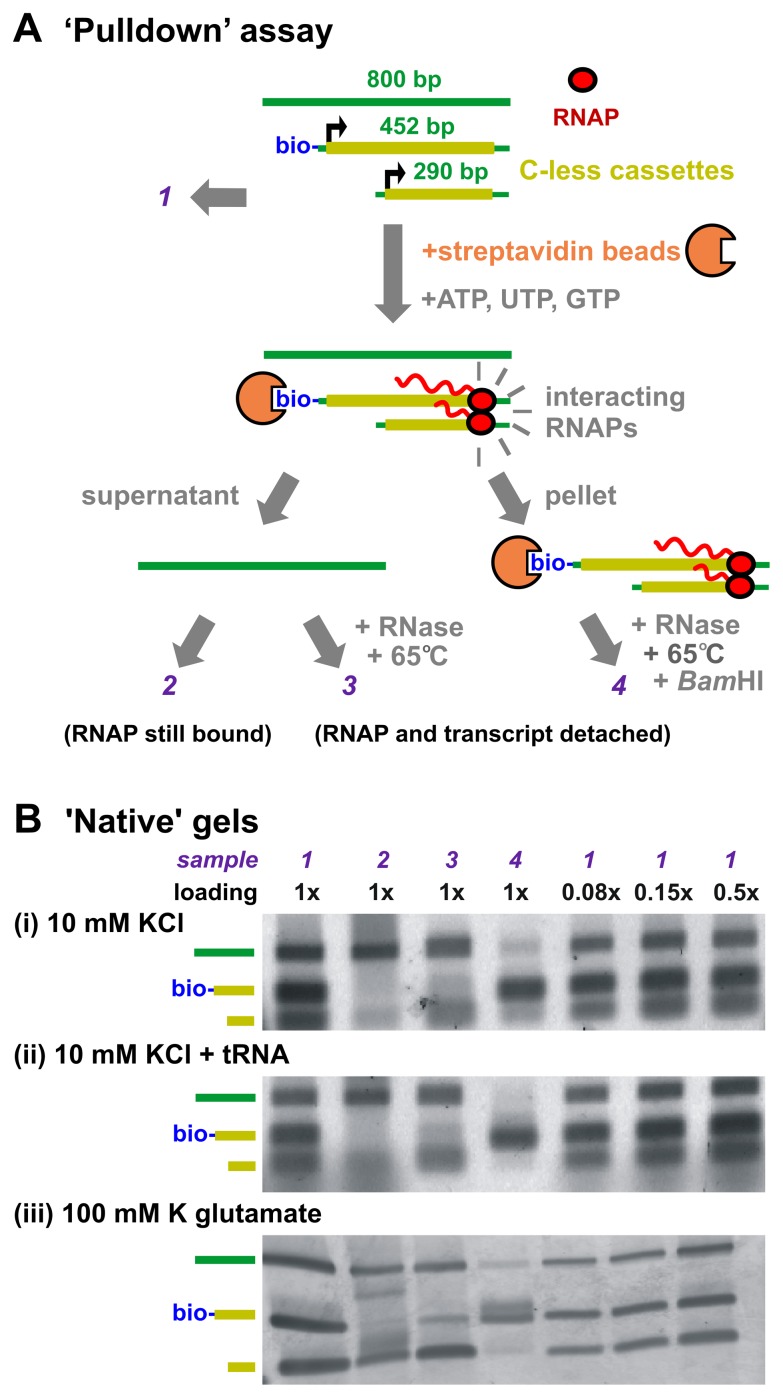
Elongation complexes do not co-purify *in vitro*. **A.** Strategy behind the ‘pulldown’ assay. T7 RNAP is mixed with three pieces of DNA (sample *1*): (i) an 800-bp promoter-less control fragment, (ii) a 452-bp template tagged with a 5′ biotin and encoding a *Bam*HI site, a T7 promoter, a C-less cassette, and a C-containing 3′ end, and (iii) a 290-bp template encoding a T7 promoter, C-less cassette, and C-containing 3′ end. After adding streptavidin beads, reactions were supplemented with ATP+UTP+GTP, and incubated for 30 s (to allow polymerases to initiate on the two templates and halt at the end of the C-less cassettes). If the now-engaged and halted polymerases interact, the 290-bp and 452-bp templates should associate (as shown). Next, beads (plus associated 290-bp and 452-bp templates) are pelleted, and the supernatant removed (sample *2*). Both supernatant and pellet are now treated with RNase and heated to 65°C to strip RNAPs and their transcripts from the templates (sample *3*); the pellet is also treated with *Bam*HI to release attached 452-bp templates from beads prior to analysis (sample *4*). If (elongating and halted) RNAPs interact (as shown), the 290-bp template (but not the 800-bp control fragment) should pellet with beads and the attached 452-bp template; then, the 290-bp template should be found in sample *4*. If they do not interact, the 290-bp template should not be found in the pellet (not shown). **B.** The assay described above was conducted in (i) 10 mM KCl (i.e., buffer LS1), (ii) 10 mM KCl plus tRNA (with 10-fold more tRNA than total template), and (iii) 100 mM potassium glutamate (i.e., buffer KGB); then, samples *1–4* were prepared, applied to ‘native’ 1.5% agarose gels, and the gels stained with SYBR green I. The 800-bp control fragment is present in samples *1–3*, but not *4* (as it fails to pellet). The 452-bp template is present in samples *1* and *4* (as it binds to beads, and pellets). Only trace amounts of the 290-bp template migrate as free DNA in sample *2* (elongation complexes migrate more slowly as a smear), but this amount is increased in sample *3* (as RNase and heat treatments release it from elongation complexes). The 290-bp template is found in sample 4 when the assay is performed in 10 mM KCl. However it is absent when the assay is performed in 10 mM KCl plus tRNA, or the more physiological buffer containing 100 mM K glutamate.

To test whether active T7 RNAPs cluster, we examined whether ECs diffusing freely in solution interacted with distinguishable ECs directly attached to beads (Fig. 1A). To achieve this, we created a transcription reaction containing RNAP as well as three DNA fragments of different lengths (Fig. S1A): a 290-bp template encoding a T7 promoter that was freely-diffusing in solution, a 452-bp template which again encoded the promoter but was bound by a biotin at its 5′ end to streptavidin-coated beads, and an 800-bp promoter-less control fragment. When ATP, UTP, and GTP (but no CTP) were added, RNAPs initiated on the two templates encoding promoters, and transcribed until they needed to incorporate CTP; they then stably halted (Fig. S1B; previous work has shown that the resulting halted ECs have half-lives >10 min; [18]).

We then isolated the ECs formed on the 452-bp templates by pelleting the beads and removing the supernatant. Any ECs formed on 290-bp templates interacting with these pulled-down ECs would then be found in the pellet. When the pelleted DNA was isolated and visualized, a small amount of the 290-bp template – but virtually no 800-bp control DNA – was found (Fig. 1Bi, sample 4). Thus it seemed that ECs on the 290-bp template were associating with the beads and being pelleted.

Examination of the DNA remaining in the supernatant using agarose gel electrophoresis allowed us to distinguish unbound templates (which migrate as free DNA) from occupied templates (which migrate more slowly; Fig. S2). When the RNAPs in the removed supernatant are stripped from their templates (by heating) before gel electrophoresis, a large amount of 290-bp template migrates as free DNA (Fig. 1Bi, sample 3). However very little 290-bp template migrates freely when RNAPs remain bound to their templates (Fig. 1Bi, sample 2). These results suggest that the majority (i.e., 60–80%) of 290-bp templates were occupied by halted RNAPs at the moment the beads were pelleted. Additional controls showed that RNAPs initiated as efficiently on the 452-bp template as on the 290-bp template (Fig. S3). Thus, we conclude that although the majority of 452-bp and 290-bp templates were occupied by RNAPs, only a small fraction of the 290-bp was pelleted.

However, we were concerned that the interaction between ECs might be caused by aggregation of nascent RNA, and not by an interaction between RNAPs. To investigate this possibility, we repeated the experiment in a buffer containing 10-fold more tRNA than DNA template (Fig. 1Bii). We expected that the tRNA would disrupt any non-specific RNA-based interactions (by competing for any RNA-binding sites), while leaving polymerase-based protein-protein interactions unaffected. When the experiment was conducted in the presence of tRNA, only tiny amounts of the 290-bp template were found in the pellet («8% of total; Fig. 1Bii, compare samples 4 and 5). Because the remaining 290-bp template did not appear to be enriched relative to the 800-bp promoter-less control fragment (Fig. 1Bii, compare samples 4 and 5), we concluded it was not pelleted due to EC-EC interactions, but rather, persisted because we only removed ∼97% of the supernatant. Our finding that no short template (or control DNA) was found in the pellet when a gentle wash step was included supports this interpretation (data not shown). Therefore, we conclude that the previously-observed interaction was based on non-specific RNA interactions. As such interactions are unlikely to be physiologically relevant (see Text S1A), we conclude that no meaningful RNAP-RNAP interactions were detected using these assay conditions.

Repeating the assay using a more physiological buffer (KGB, which contains 100 mM K glutamate, instead of LS1, which contains 10 mM KCl) yielded a similar conclusion even though no tRNA was present: although most templates were occupied by RNAPs (Fig. 1Biii, compare free-migrating short template in samples 2 and 3), no enrichment of the 290-bp template relative to the control DNA was observed (Fig. 1Biii, sample 4). Identical results were obtained when the total concentration of ECs was increased to 0.1 µM, and when bovine serum albumin was used as a blocking agent instead of casein (data not shown).

Were ECs to form stable, oligomeric clusters, we would expect that most of the occupied short template (i.e., ∼60–80% of total) would interact with the bead-bound ECs, and so be found in the pellet. Our finding that less than a few percent of the short templates are pulled down therefore supports the conclusion that ECs do not form stable clusters under these conditions.

### T7 RNAP ECs do not interact with a K_d_<1 µM

In our previous experiment, we found that ECs attached to beads were unable to ‘pull down’ ECs in solution. However, it is possible that the pelleting of the bead-bound ECs disrupted their interaction with ECs in solution.

To eliminate this possibility, we used fluorescence correlation spectroscopy (FCS) to study EC diffusion behaviour. In this non-perturbative technique, a laser is focused on a ‘confocal spot’ in solution, allowing the measurement of the diffusion times – and therefore relative sizes – of fluorescently-labelled ECs [28]. Since diffusion is slower for larger complexes, diffusion times increase with complex size. We expected single ECs with no interaction partners to diffuse relatively quickly, with a small diffusion time less than or equal to the sum of the diffusion times of their components (i.e., an RNAP and its template; Text S1B); in contrast, interacting ECs should diffuse more slowly as large complexes containing multiple RNAPs and templates – with diffusion times greater than those expected for non-interacting ECs.

**Figure 2:**
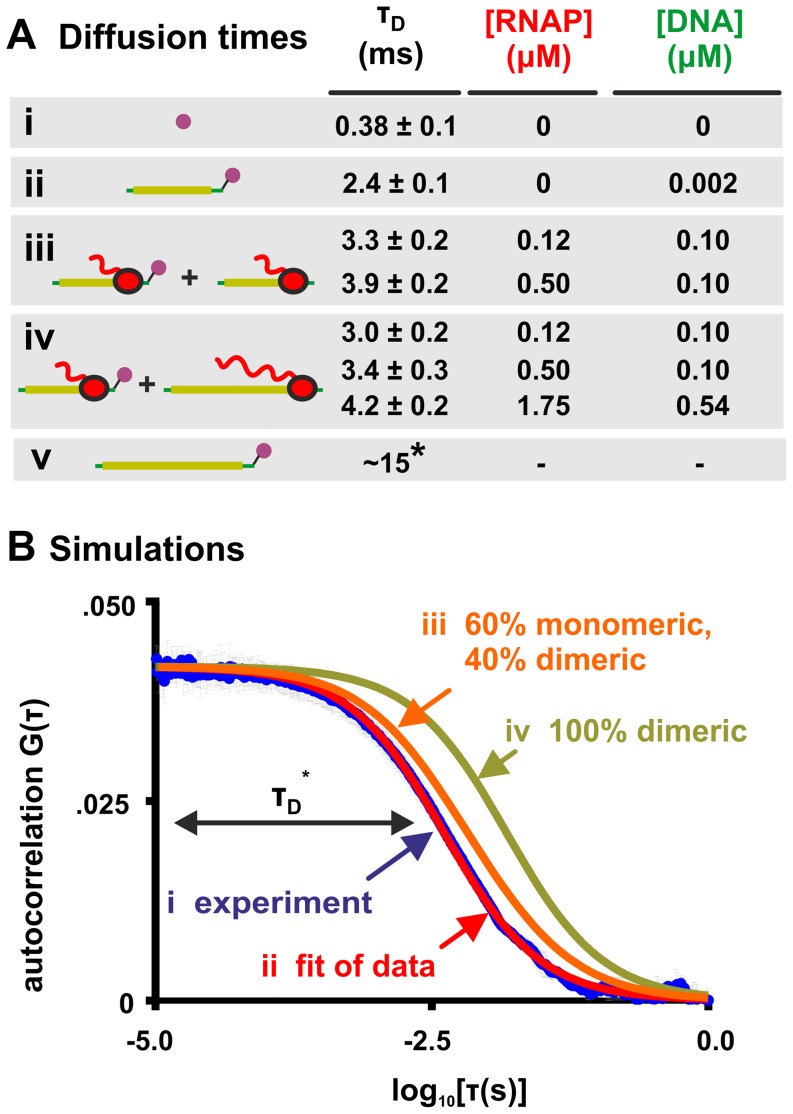
Elongation complexes do not interact *in vitro* with a *K_d_*<1 µM. **A.** The diffusion times (*τ_D_*) of different fluorescently-labeled molecules (all at 2 nM; fluorophores indicated by purple circles) in buffer LS1, as determined by FCS using a single-species model. (i) Rhodamine 6G alone. (ii) A 70-bp template containing a T7 promoter, a 23-bp C-less cassette, and a C-containing 3′ end labeled with Cy3B. (iii) T7 RNAP ECs. A reaction containing labeled (2 nM) and unlabeled (0.1 µM) 70-bp templates was initiated by the addition of ATP+UTP+GTP, and incubated for 30 s to allow RNAPs to initiate on the templates and halt at the first C residues; then, the average diffusion time of the labeled templates was measured. (iv) As in (iii), except the unlabeled 70-bp template is replaced by an unlabeled 452-bp template encoding a T7 promoter, a C-less cassette, and a C-containing 3′ end (at 0.1–0.54 µM). This replacement does not significantly change the diffusion time of the labeled ECs, suggesting that they do not interact with unlabeled ECs. (v) Estimated diffusion time of the 452-bp template alone (Text S1C). For all *τ_D_* values, error was calculated using standard deviation (n≥3). **B.** Expected RNAP clustering. (i) An autocorrelation curve measured in the experiment of Fig. 2Aiv (template and RNAP concentrations were 0.54 µM and 1.75 µM). Error bars represent standard deviation (n = 3). (ii) A fit of (i) using a single species model (Eq. 1); *τ_D_* = 4.2 ms. (iii) The calculated autocorrelation function one would observe in the experiment (i), if RNAPs interacted with a *K_d_* of 1 µM (calculated using a two-species model, Eq. 2). Sixty percent of labeled ECs diffuse freely with *τ_D_* = 4 ms, while 40% are in RNAP dimers containing a 452-bp template, and so have a *τ_D_* of 15 ms. This curve yields a *τ_D_* of 6.0 ms when fit using a single-species model, and is clearly distinguishable from the measured data of (i). (iv) The autocorrelation function one would expect to observe in the experiment (i), were all labeled ECs to interact with a 452-bp template; all complexes have a *τ_D_* of 15 ms.

We began by calculating an expected diffusion time for non-interacting ECs. We determined that the diffusion time of the 70-bp fluorescently-labeled template upon which our ECs would be formed was 2.4±0.1 ms (Fig. 2Aii). This measurement was in agreement with values determined previously (Text S1C). We then calculated that T7 RNAP would – because of its size and globular nature – have a diffusion time of 2–3 ms (Text S1C). Assuming that the diffusion time of a complex would be less than the sum of the diffusion times of its parts, we concluded that non-interacting ECs would have a diffusion time of 2.4–5.4 ms. If ECs had a diffusion time above this range, it would suggest the existence of larger, and therefore higher-order, complexes.

To generate ECs that could be tracked by FCS, we allowed RNAP to initiate on a 70-bp fluorescently-labeled template in the presence of ATP, UTP, and GTP. Under these conditions, the enzyme produced a 23-bp transcript before stably halting when the first C needed to be incorporated (Fig. S1). The majority of such a short nascent transcript is hidden within the RNAP (or bound to its surface; [27]), and we anticipated that the few bps emerging from the EC would not drive the RNA-based interactions observed in our ‘pulldown’ assay.

We expected that the templates in the EC-containing solution would be found in one of three populations: unoccupied templates, templates incorporated into ECs that are not bound to other ECs, and templates incorporated into ECs which in turn are bound to other ECs. For complexes with diffusion times within an order of magnitude of one another, FCS essentially reports the average diffusion time of all fluorescent species; thus fast-diffusing templates not bound to clustered RNAPs could – if numerous enough – easily obscure the existence of more slowly-diffusing EC clusters. To ensure that the fraction of templates not incorporated into ECs was negligible, we used more RNAP than template in our reactions, and performed extensive controls to show that virtually every template was bound by an active RNAP (Text S1D).

The fraction of ECs found in clusters depends upon the strength of the attraction between RNAPs; as most protein-protein interactions have *K_d_* between 1 nM and 1 µM [29], we expected that the strength of any EC clustering would also fall within this range. To detect such interactions, we required EC concentrations >0.1 µM; unfortunately, our FCS setup could only measure fluorescent species present at concentrations below 50 nM. To allow higher concentrations of ECs, we used a low concentration of labeled template (always 2 nM) and a large excess of unlabeled template (up to 0.54 µM) in our transcription reactions. ECs formed on unlabeled templates would not be directly visible to our FCS assay, but could still bind to the labeled ECs and so retard their diffusion.

After initiating a transcription reaction containing 2 nM labeled 70-bp template, 100 nM unlabeled 70-bp template, and 120 nM RNAP, we measured the average diffusion time of the now-occupied templates to be 3.3±0.2 ms (Fig. 2Aiii). To be absolutely confident that all templates were incorporated into ECs (Text S1D), we repeated the experiment using an increased RNAP∶template ratio of 5∶1; the template diffusion time marginally increased to 3.9±0.2 ms (Fig. 2Aiii).

These diffusion times fall squarely within the range expected for non-interacting ECs, and thus provide no evidence for RNAP clustering. However, we were unable to calculate precisely an expected diffusion time for small EC clusters (e.g., dimers or trimers), and thus could not formally exclude the possibility that our ECs were diffusing as dimers or other lower-order complexes, rather than monomers.

To set a lower limit on the diffusion times of EC clusters, we replaced the 70-bp unlabeled templates in our experiment with 452-bp unlabeled templates (Fig. 2Aiv; S1). Under these conditions, any EC clusters would contain at least one EC formed on a 452-bp template, and so would possess a **T**_D_>15 ms (i.e., the diffusion time of the 452-bp template alone; Fig. 2Av; Text S1C). However, substituting unlabeled 452-bp templates for unlabeled 70-bp templates had no significant effect on the diffusion time of the labeled 70-bp ECs, which still diffused with **T**_D_ = 3–4 ms (Fig. 2Aiii–iv). This was the case even when the concentration of occupied 452-bp templates was increased to 0.54 µM (Fig. 2Aiv). We conclude that – under our assay conditions – the overwhelming majority of RNAPs halted on the labeled 70-bp templates did not bind to the RNAPs halted on the 452-bp templates. We note that our finding that the diffusion times of ECs was relatively unaffected by the ratio of RNAP∶template is not consistent with the possibility that an interaction was present, but titrated out by excess RNAP.

To estimate the detection limit of our assay, we calculated the autocorrelation function that our assay would have produced, if the halted RNAPs were to interact. In the experiment of Figure 2Aiv, we measured the autocorrelation function of 2 nM labeled ECs (formed on 70-bp templates), in the presence of 0.54 µM unlabeled ECs (formed on 452-bp templates). If ECs dimerized with *K_d_* = 1 µM, such a solution would contain ∼40% dimers and ∼60% monomers. We calculated the autocorrelation function of this solution by conservatively modeling monomers (70-bp templates bound by halted RNAPs) as having a τ_D_ of 4 ms, and dimers (complexes containing two active RNAPs, one 70-bp template, and one 452-bp template) as having a τ_D_ of 15 ms. We find that such a solution would produce an autocorrelation function clearly distinguishable from the one measured in the experiment summarized in Fig. 2Aiv (with results in Fig. 2B). Thus, we conclude that – under our *in vitro* conditions – active T7 RNAPs do not interact with a *K_d_*<1 µM.

### Genes transcribed by T7 RNAP do not detectably interact

To test whether ECs interact in their native cellular environment (i.e., in living *E. coli*), we used ‘chromosome conformation capture’ (3C; [30]) to determine whether or not two T7 promoter-encoding genes – which are located far apart on the bacterial chromosome – are in contact more frequently when transcribed by T7 RNAP. If ECs active at different genomic sites interacted, we expected that their respective transcription units would also be brought into close proximity.

**Figure 3:**
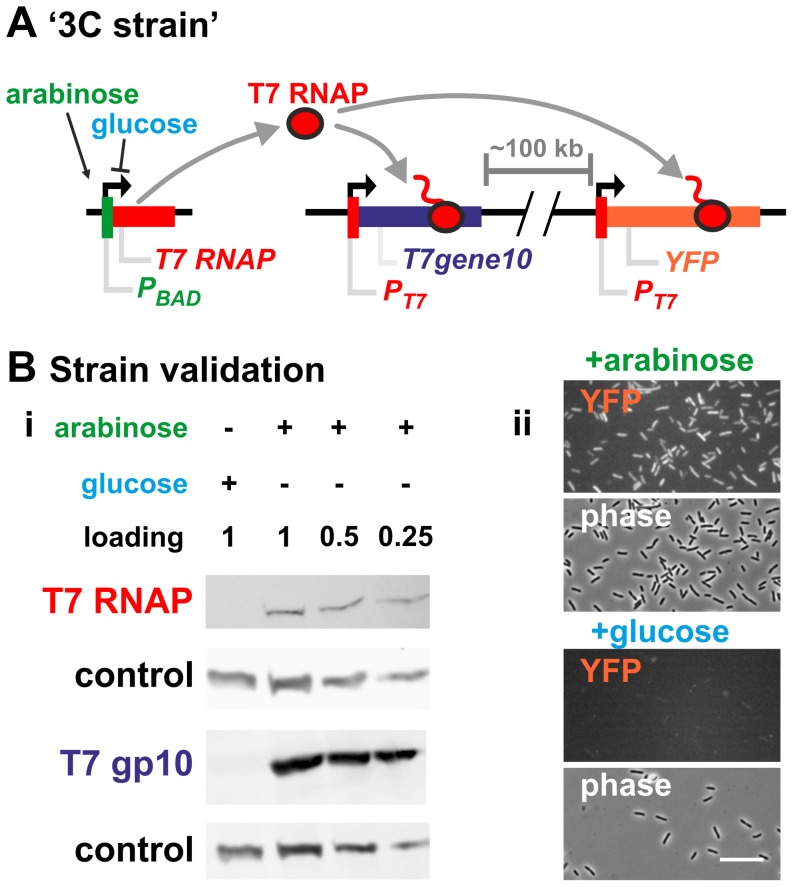
Validating the ‘3C strain’. **A.** Diagram of the ‘3C strain’ genome. The strain expresses T7 RNAP (under control of a P_BAD_ promoter) in the presence of arabinose, but not in the presence of glucose. T7 RNAP then transcribes two transgenes driven by T7 promoters – *T7gene10* and *YFP* – integrated 100 kb apart in the bacterial chromosome. If active T7 RNAP clusters, these transgenes should interact in the presence of arabinose (i.e., when transcribed) but not glucose (i.e., when inactive). **B.** Validating ‘3C strain’. (i) Cells were grown to OD_600_ = 0.4 in media containing arabinose or glucose, and their protein content analyzed using SDS-PAGE and western blotting. Probing for T7 RNAP shows it is expressed in the presence of arabinose (induction) but not glucose (repression). Probing for T7gp10 shows the same pattern, confirming that the corresponding gene is transcribed by T7 RNAP. In both blots, NusA is used as a loading control. (ii) Cells from the ‘3C strain’ imaged using fluorescence microscopy. YFP is detected when cells are grown in the presence of arabinose (+T7RNAP) but not glucose (−T7RNAP), confirming that its gene is transcribed by T7 RNAP. Both YFP images have the same intensity scale. Bar: 20 µm.

We began by constructing a strain that would allow us to test this hypothesis. We first inserted two genes encoding T7 promoters (*P_T7_-YFP* and *P_T7_-T7gene10*) into the *E. coli* genome 100 kbp apart (Fig. 3A). We expected that if ECs clustered, these two genes would be brought into contact when transcribed by the T7 polymerase. To control the levels of T7 RNAP in the cell, we integrated a gene expressing the polymerase under the control of a P_BAD_ promoter (Fig. 3A). This gene produced high levels of T7 RNAP when cells were grown in arabinose, but negligible levels when cells were grown in glucose (Fig. 3Bi). Controls confirmed that this T7 RNAP efficiently transcribed the two T7 promoter-driven test genes (Fig. 3B).

We then used ‘3C’ to determine whether or not the two test-genes were in contact more frequently when transcribed by T7 RNAP. This PCR-based method determines the relative interaction frequencies of different genomic regions *in vivo* [30]. Cells are fixed with formaldehyde, and their chromatin digested with a restriction enzyme. Cross-linked restriction fragments are then ligated together, and the frequency of ligations between different pairs of restriction fragments is measured by PCR.

**Figure 4:**
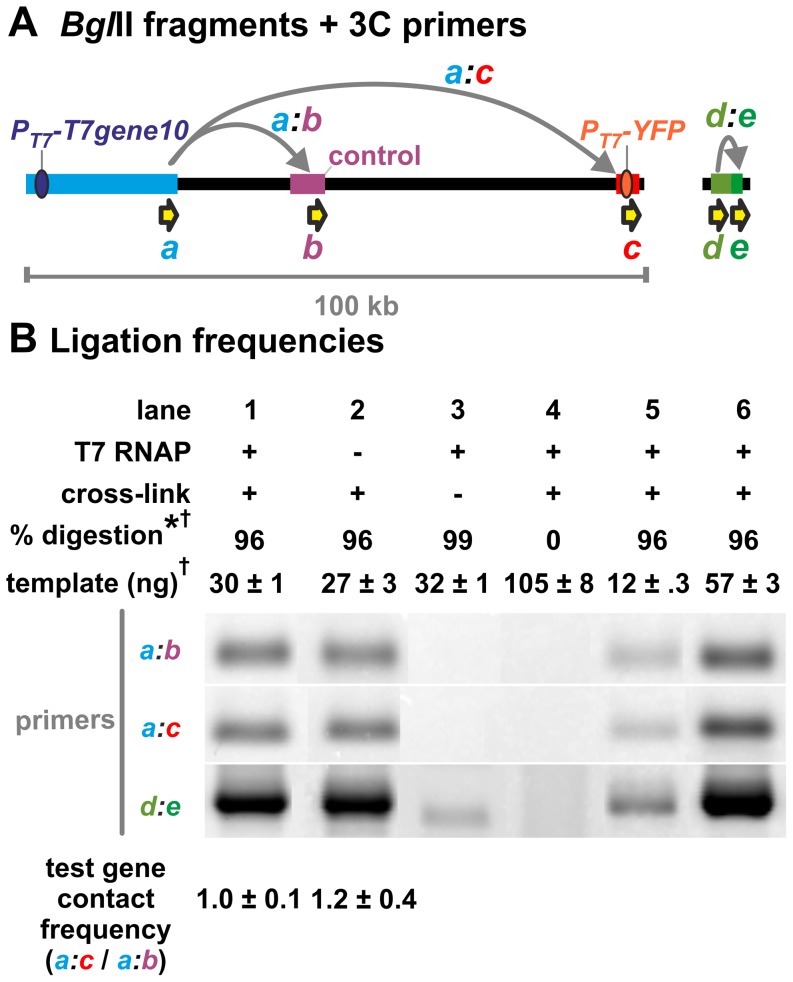
Genes transcribed by T7 RNAP do not interact *in vivo*. **A.** Diagram of *Bgl*II fragments (thick coloured regions), PCR primers (yellow arrows) and ligation products (grey arrows) used in 3C. Ovals denote transcription units under the control of the T7 promoter. We determined the ligation frequency of the *Bgl*II fragment encoding *P_T7_-T7gene10* (light blue; 24 kb) with (i) the 3-kb fragment encoding *P_T7_-YFP* located 80 kb away (orange; using primers *a* and *c*), and (ii) a 5-kb promoter-less control fragment 22 kb away (purple; using primers *a* and *b*). As a secondary control for 3C efficiency, we also measured the ligation frequency of two adjacent fragments located at a randomly-chosen genomic site (pink and green; using primers *d* and *e*). This genomic map is drawn to scale. **B.** 3C. The ‘3C strain’ in Fig. 3A was grown to OD_600_ = 0.4 in arabinose (+T7 RNAP) or glucose (−T7 RNAP), 3C templates prepared using *Bgl*II, and ligation products detected by PCR using the primer pairs indicated. Images show PCR products resolved on an agarose gel, and stained with SYBR green I. Loading controls (lanes 5–6) show that band intensities are proportional to the amount of ligation product present in the preceding PCR reactions, and thus to the contact frequency of the selected restriction fragments. All PCR products are of the expected size, and depend upon formaldehyde crosslinking (lane 3) and restriction nuclease digestion (lane 4). For each condition, the ‘test gene contact frequency’ (of *T7gene10* with *YFP*) was calculated by dividing the intensity of the band produced by the primers *a:c* by the intensity of the band produced by primers *a:b*. This adjustment corrected for differences in 3C efficiency, and so allowed ligation frequencies obtained from different samples (i.e., ± T7 RNAP) to be directly compared. Transcription of *T7gene10* and *YFP* by T7 RNAP had no effect on their contact frequency (compare test gene contact frequencies in lanes 1 and 2). Normalizing for 3C efficiency using the ligation frequency of the adjacent fragments (i.e., *a:c*/*d:e*) yielded the same result (1.9±0.6 a.u. for +T7 vs. 2.7±0.2 a.u. for −T7). a.u. = arbitrary units. *all measurements had standard deviations of less than 1% (n = 3). ^†^assessed by qPCR.

We performed 3C on cells grown in either arabinose or glucose, and – under both conditions – determined the frequency with which the *Bgl*II restriction fragment containing *P_T7_-T7gene10* was ligated to the fragment containing *P_T7_-YFP* (Fig. 4A). We found that transcription of the two test-genes by T7 RNAP had no effect on the ligation frequency of their respective restriction fragments (Fig. 4B, lanes 1,2, primer pair *a:c*). Controls showed that the formation of the ligation products depended on formaldehyde crosslinking (Fig. 4B lane 3), and that the efficiency of the 3C protocol was independent of the presence of T7 RNAP (Fig. 4B primer pairs *a:b*, *d:e*). We conclude that if T7 RNAP ECs do interact, they do not do so strongly enough to significantly change the contact frequency of our transgenes.

## Discussion

Many RNAPs co-associate when active; this clustering often influences function, for example, by increasing activity (see Introduction). In order to determine whether T7 RNAP behaves similarly, we used three independent assays to test whether this polymerase also clusters when active. In the first assay, we attempted to ‘pulldown’ ECs in solution using ECs attached to beads (Fig. 1A), and found no evidence for a direct protein-protein interaction (Fig. 1B). As this assay required physical manipulation of ECs which might break weak EC-EC interactions, we performed a second assay using fluorescence correlation spectroscopy; this directly measures complex sizes without the need for physical manipulation, but it also failed to provide evidence for clustering (Fig. 2). Therefore, if T7 ECs do interact *in vitro*, it seems likely that they will do so with a *K_d_* outside the detection range of our assays (i.e., >1 µM, which is much greater than the estimated *in vivo* concentration of 30 nM; see Text S1G). As the buffers and enzyme concentrations we use are typical of those widely applied by others [18], [20], [24], we conclude that in the majority of the instances where it has been studied, T7 RNAP has behaved as a monomer.

Because interactions present *in vivo* can be missed by *in vitro* assays (e.g., if they require macromolecular crowding, or a ‘bridge’ protein), we also used chromosome conformation capture (3C) to examine association *in vivo* (Fig. 3). In mammals, 3C readily detects RNAP-driven clustering of active genes [31], [32], even when those interactions occur in only ∼1% cells in the population [31]. However, 3C failed to provide any evidence for clustering in bacteria (Fig. 4), even though the genes we examined are probably as tightly packed with polymerases as the ribosomal cistrons (our T7 RNAP-based expression system can produce as much RNA as all seven ribosomal cistrons combined, which are each typically occupied by 70 RNAPs/gene; [33], [34]; see also Text S1E).

However, our 3C assay does have limitations. It involves formaldehyde fixation, which can rapidly disrupt nucleoid structure [35], [36], and so could – in principle – also destroy any clustering. Note, however, that clustering of genes binding H-NS, a global transcriptional silencer, can be detected by 3C [37]. We may also have inadvertently inserted our two test genes in regions of the bacterial genome that interact rarely. Another problem is that the phage-encoded proteins expressed during T7 infection were not present in our 3C assay. Any EC clustering dependent upon a phage-encoded ‘bridge’ protein would not have been detected in our assays (this, and other potential problems are discussed in Text S1F).

In conclusion, we find no evidence for the clustering of active forms of T7 RNAP either *in vitro* or *in vivo*. Our *in vitro* assays allow us to exclude the possibility of a strong interaction between ECs (i.e., with *K_d_*<1 µM). Our *in vivo* 3C assay does not allow us to draw equally firm conclusions, but nevertheless suggests that if an interaction does exist, it is likely to be weak, disrupted by our assays, or dependent on phage proteins not present in our 3C experiment. If an interaction does not exist, then the phage enzyme clearly has different properties from its mammalian counterparts, with which it shares only minimal structural homology [38]. But, then, Nature must find other ways of immobilizing the phage enzyme, or otherwise preventing the entanglement of nascent transcripts about their templates [11], [39].

## Materials and Methods

### Templates

Template DNA was created by PCR from pLSG407 [40] unless otherwise indicated. KRF3/28 was the product of a PCR using primers KRF3 and KRF28. The ‘452-bp template’ (created using KFR3/28 as a template) was the product of primers KRF28 and KRF32, and contained a 5′ biotin, followed by a *Bam*HI site, a T7 promoter, and a 382-bp C-less cassette followed by 16 bp of C-containing DNA. The ‘290-bp template’ contained a T7 promoter followed by a 243-bp C-less cassette and 12 bp of C-containing DNA, and was the product of primers KRF36 and KRF37. The ‘70-bp template’ was created using the oligonucleotide template KRF47 in combination with the primers KRF42 and KRF45, and contained a T7 promoter followed by a 23-bp C-less cassette and 12 bp of C-containing DNA. Template DNA was purified using a Minelute PCR purification kit (Qiagen).

### Labeling of DNA with fluors

The fluorescently-labelled 70-bp DNA template was prepared in the same manner as the unlabeled template, except that the primer KRF43 was replaced by the fluorescently-labeled primer KRF45 (see Table S1 for primer sequence). KRF45 contained an amine-labeled dT residue near its 5′ end, and was labeled using succinimidyl esters of Cy3B (GE Healthcare) or Atto647 (Atto-Tec) following the manufacturer's instructions. One hundred micrograms of KRF45 was dissolved in 100 µL of H_2_O and extracted three times with an equal volume of chloroform. After the addition of 10 µL 3 M sodium chloride and 250 µL ethanol, the oligonucleotide was incubated at −20°C for 30 min, and then centrifuged at 12,000 * g for 30 min at 4°C. The pellet was allowed to dry, resuspended in 75 µL of 0.1 M sodium borate (pH 8.5), and frozen in 25 µL aliquots. A 50 nmol aliquot of succinimidyl ester was then resuspended in 5 µL DMSO, mixed with a 25 µL aliquot of KRF45, and left overnight (in darkness) at 25°C. Labeled oligonucleotides were purified away from unconjugated fluorophore by ethanol precipitation, followed by one wash with 70% ethanol. Comparing the absorbance of the oligonucleotide at 260 nm (using ε_260_ = 193,750 M^−1^cm^−1^) with its absorbance at 563 nm (for Cy3b; using ε_563_ = 130,000 M^−1^cm^−1^, CF_260_ = 0.08) or 650 nm (for Atto647N; ε_650_ = 150,000 M^−1^cm^−1^,CF_260_ = 0.06) showed that 90–100% of oligonucleotides were labeled. Denaturing urea-PAGE followed by visualization of the unstained gel with a FLA5000 imager showed that >90% of the dye migrated with the purified oligonucleotide.

### ‘Pulldown’ assay

The transcription buffer used in this experiment was either low-salt buffer (LS1; 40 mM Tris-acetate pH 7.6, 10 mM potassium chloride, 15 mM magnesium acetate, 5 mM dithiothreitol, 0.1 mg/mL N,N-dimethylated casein, 0.05% Tween 20, 0.4 U/µL RNase inhibitor, Roche) or the more physiological potassium-glutamate buffer (KGB; 40 mM Tris-acetate pH 7.6, 100 mM potassium glutamate, 15 mM magnesium acetate, 5 mM dithiothreitol, 0.1 mg/mL N,N-dimethylated casein, 0.4 U/µL RNase inhibitor; [41]). The buffer LS1 was used because a study of the effect of buffer composition on T7 RNAP activity found this formulation to be optimal [24]. The buffer KGB was used because it is thought to mimic the cellular milieu [41]. The blocking agent in KGB was changed from bovine serum albumin (BSA) to casein because the latter yielded slightly higher T7 RNAP activity [24]. The experiment was performed at 25°C (when LS1 was used) or 37°C (when KGB was used).

A 60 µL transcription reaction contained transcription buffer plus 4 pmol His_6_-tagged T7 RNA polymerase, 0.6 pmol biotinylated 452-bp template, 0.6 pmol 290-bp template, and 0.2 pmol 800-bp control DNA. Two samples (2 µL each) were taken, and immediately added to 10 µL ice-cold 1× TBE loading dye (89 mM Tris-borate, 89 mM boric acid, 2 mM EDTA, 0.05% bromophenol blue). Separately, 30 µL of M270 magnetic streptavidin beads (6.7×10^8^ beads per mL; Invitrogen) were washed twice in 200 µL transcription buffer, and then resuspended in the remaining 56 µL of the transcription reaction. After incubation for 20 min (with mixing after 10 min), ATP, UTP, and GTP were added to a final concentration of 0.5 mM. Then, after 30 s, beads were pelleted with the aid of a magnet, and the supernatant removed. After removing a 2 µL sample (and addition to TBE loading dye as above), supernatants were heated to 65°C for 10 min, and treated with 10 U RNase I (Promega) for 10 min at 37°C. The pellet was resuspended in water, then 10× LS1 was added to a final concentration of 1×, followed by the addition of 10 U/µL RNase I and 10 U *Bam*HI (assuring the initial ∼60 µL volume was conserved). After 20 min at 37°C, beads were pelleted, the supernatant heated to 65°C for 10 min, and 2 µL samples collected (and added to TBE loading dye as above).

### Fluorescence correlation spectroscopy

Transcription reactions (performed in LS1) were initiated by addition of ATP, UTP, and GTP to 0.5 mM, and incubated for 30 s at 25°C before being pipetted onto a cleaned coverslip at 25°C. Fluorescence correlation spectroscopy was performed as described [42]. Time traces were acquired for 10 s using a SPQR-14 avalanche photodiode (Perkin Elmer), and autocorrelation functions were produced in real-time using a Flex02-02D correlation card (Correlator.com).

As our setup has a large pinhole, and therefore an elongated confocal spot (longitudinal radius, *w_z_*>>*w_xy_*, the axial radius), translational diffusion times (*τ_D_*) were extracted from autocorrelation curves by fitting to a two-dimensional single-species model, 
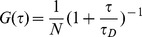
 (equation 1; [43]), where τ is the delay time, G(τ) is the autocorrelation function, and *N* is the mean number of fluorescent molecules in the observation volume over the measurement. Experimentally acquired FCS curves were fit very well by this model (e.g., Fig. 2B and Fig. S4). Although the molecules we analyze diffuse in three dimensions, the 3D model, 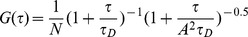 (where *A* = *w_z_/w_xy_*; equation 2; [28]), simplifies to the two-dimensional model (equation 1) in the case of an elongated confocal spot [44]. To ensure that the 2D model was appropriate for modeling our data, we fit our Rhodamine 6G autocorrelation curves with both the 2D and 3D models. Fitting the data with the 3D model did not significantly change the values we obtained for either *τ_D_* or *N*, however *A* could not be fit with reasonable confidence intervals; changing the value of *A* therefore did not substantially affect the goodness of fit, a behavior consistent with confocal volumes where *w_z_*>>*w_xy_*. To ensure that our choice of model did not change the conclusions of our FCS work, we re-fit all of our FCS curves (i.e., all the data in Fig. 2A) using the 3D model and setting *A* = 7, a common value for single-photon excitation setups; doing so increased all *τ_D_* values by a small amount (∼3–5%), with the difference between any two *τ_D_* values changing by not more than 2%.

Two-species curves were calculated using the model 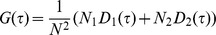, where 
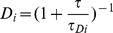
, and *N_1_* and *N_2_* are the mean number of fluorescent molecules of species 1 and 2, respectively, in the observation volume (equation 3; [28]). Curve fitting was performed in MATLAB (Mathworks). These models were also used to calculate the curves in Figure 2B.

Fluorescence fluctuations were unlikely to be the result of dye-specific or photoinduced-photophysics, as the fitted N and τ_D_ of the fluorescently-labelled 70-bp template were unchanged when Atto647N was substituted for Cy3B, or when laser power was increased 10-fold (data not shown).

In order to convert diffusion times (which depend on the size of the observation volume generated by the FCS setup) into diffusion coefficients (which are physical constants), we calculated the radius of the observation volume, ω, using 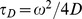 (equation 4; [28]). Measuring a diffusion time of 0.38±0.1 ms (fitting to equation 1) for the fluorescent standard rhodamine 6G (*D* = 4.14·10^−6^ cm^2^/s; [45]) allowed us to calculate *ω* = 780±100 nm. This observation volume is slightly larger than usual in order to maximize the number of photons captured from fluorophores during single-molecule FRET experiments carried out on the setup; however, this does not affect our ability to measure diffusion times.

### Chromosome conformation capture

This protocol – modified from the original [30] for use in bacteria – was generously provided by Mark Umbarger (Harvard; [46]). The *E. coli* strain KF22-1 was grown overnight to saturation in LB+50 µg/mL kanamycin, diluted by 1∶250 into flasks containing 25 mL of the same media (preheated to 37°C), and incubated at 37°C with shaking. After 30 min, arabinose was added to 0.4%, or glucose was added to 0.2%. When the cultures reached an OD_600_ of 0.4, sodium phosphate (pH 7.6) and formaldehyde were added to final concentrations of 10 mM and 1% respectively (except for non-crosslinked controls). After 20-min incubation at 37°C and 20-min incubation in an ice bath (both with light shaking) the formaldehyde reactions were quenched by addition of glycine to 0.125 M, and incubated for 5 min at 25°C. All cultures were then spun down at 5000 * g for 10 min, washed once with 50 mL ice-cold Tris-buffered saline (20 mM Tris-HCl pH 7.5, 150 mM NaCl), pelleted, and stored at −80°C.

The pellets were then resuspended in 1 mL TE buffer (10 mM Tris, 1 mM EDTA, pH 8), and minor adjustments were made to assure that the OD_600_ of all samples was equal. For each pellet, 60 kU of Ready-Lyse Lysozyme (Epicentre) was added, and the mixture incubated at 25°C for 15 min with occasional gentle pipetting to resuspend cells. SDS was then added to a final concentration of 0.5% and cells were allowed to incubate for 30 min.

Five microlitres of solubilized chromatin (∼100 ng DNA) were mixed into a 50 µL volume containing 1× restriction buffer #3 (New England Biolabs) and 1% Triton X-100, and incubated for 20 min to allow the Triton to neutralize the SDS. Fifty units of *Bgl*II (New England Biolabs) were added, and the chromatin digested for 2.5 h at 37°C with light shaking. One additional sample served as a no-restriction enzyme control. The reaction was then halted by addition of SDS to 1%.

In order to form intra-molecular ligation products, 60 µL digested chromatin was added to 760 µL ‘ligation mix’ containing 1× T4 ligase buffer, 1 mM ATP, 25 µg/mL BSA, 1% Triton X-100, and 2.4 kU/mL T4 DNA ligase. One additional sample served as a ‘no ligase’ control. Ligase mixtures were then incubated for 16°C for 1 hr. The reaction was halted by the addition of EDTA to 10 mM, and incubated overnight with 50 mg of proteinase K at 65°C. Four hundred microlitres of the DNA solution was then extracted twice with 400 µL of 25∶25∶1 phenol∶chloroform∶isoamyl alcohol. Glycogen was added to a final concentration of 50 µg/mL. Ice-cold sodium acetate and ethanol were then added to final concentrations of 0.75 M and 70% (v/v) respectively. The DNA-glycogen mixture was incubated at −80°C for 3 h, and then spun down at 20,000 * g at 4°C for 20 min. The pellet was then washed with 1 mL 70% (v/v) ethanol (25°C), air dried, and resuspended in 12 µL distilled, deionized, H_2_O.

PCR was performed using FlexiTaq DNA polymerase (Promega) and 1× reaction buffer, 1.75 mM MgCl_2_, 0.2 mM dNTPs, 0.4 µM primers and 2% DMSO on a thermocycler using the following program: (i) 95°C for 1 min, (ii) 95°C for 1 min, (iii) 65°C for 45 s, (iv) 72°C for 2 min, (v) repeat steps ii–iv 35 times, and (vi) 72°C for 6 min.

Ligations between restriction fragments 1 (T7 gene 10) and 8 (control DNA fragment) were amplified using primers KF101to8BglIIfw and KF101to8BglIIrv; these primers were designed to produce a fragment of 243 bp (this corresponded to ligation product *a:b* in Fig. 4A; all primer sequences can be found in Table S1). Ligations between restriction fragments 1 (T7 gene 10) and 16 (pT7-Ypet) were amplified using primers KF101to16BglIIfw and KF101to16BglIIrv; these primers were designed to produce a fragment of 217 bp (this corresponded to ligation product *a:c* in Fig. 4A). We queried the inversion and ligation of two adjacent fragments of *E. coli* genomic DNA by PCR using primers 3CposconA and 3CposconB; these primers were designed to produce a fragment of 443 bp (this corresponded to ligation product *d:e in* Fig. 4A). The identity of all PCR products was confirmed by measuring the size of the products, and by digesting these products with *Bgl*II (data not shown).

We quantified the amount of ligation products produced in our 3C reactions using PCR, following well established protocols [47]. We began by optimizing PCR conditions (i.e., amount of 3C template per reaction, and number of PCR cycles) to ensure that the amount of PCR product produced was linearly related to the amount of ligation product initially present in the PCR reactions. This was accomplished by performing PCR reactions containing serial dilutions of the 3C template, subjecting the PCR reactions to gel electrophoresis (on a TBE-2% agarose gel), staining the gels with SYBR green I, and measuring the intensities of the bands corresponding to the amplification products (using AIDA image analysis software). We found that, for all the ligation products we examined, 36 PCR cycles on 30 ng of our 3C template resulted in bands with an intensity that was proportional to the amount of ligation product in the initial PCR reactions (e.g, see Fig. 4B lanes 1, 5, and 6).

Using these conditions, we then conducted PCR on all experimental samples in triplicate. For each primer pair, controls containing 15 ng and 60 ng ‘+T7’ 3C template (i.e., 0.5× and 2× the normal amount) were also included to ensure that the intensity of the bands produced on our gels was linearly related to the amount of ligation products in the PCR reactions (these controls are found in Fig. 4B, lanes 1, 5 and 6). Only samples run on the same gel were directly compared.

The goal of the experiment was to determine whether the interaction frequency of the transgenes P_T7_-gene10 and P_T7_-YFP, (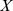), in the presence of T7 RNAP, 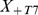, was greater than the interaction frequency of these two genes in the absence of T7 RNAP, 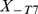. In other words, the goal was to determine whether 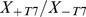 was greater than 1. The relationship between interaction frequencies (which occur in the cell) and ligation frequencies (which are present in a 3C template sample) is given by 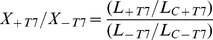 (equation 5), where 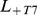 and 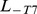 are the ligation frequencies of the transgenes in the presence and absence of T7 RNAP, while 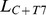 and 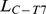 are the ligation frequencies of two control restriction fragments that should interact at the same rate regardless of whether or not the transgenes are transcribed by T7 RNAP (these two control ligation products were amplified by primers *a:b* or *d:e*; Fig. 4A). This equation states that directly comparing ligation frequencies between different 3C samples is possible only after differences in the efficiency of the 3C protocol between samples are controlled for.

If we assume that the intensity of the band produced by each amplified ligation product is proportional to the original amount of ligation product in the 3C template (we do, indeed show that this is the case, see above, and Fig. 4B lanes 1, 5, and 6), then the intensity of the band seen in the gel, 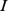, is related to the amount of ligation product in the PCR reaction, 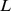, by 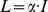, where 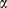 is the efficiency of the relevant primer pair. Then 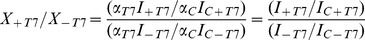 (equation 6). This equation reveals that because the experiment is ultimately interested in a change in a single interaction frequency, primer efficiencies cancel out, and have no effect on the final result. It also gives the expressions that must be measured in order to determine whether the interaction frequency of the two transgenes changes in the presence of T7 RNAP. The values of 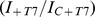 and 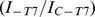 are given by the ‘test gene contact frequencies’ in Fig. 4B lanes 1 and 2. Because these values are virtually identical, 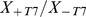 is ∼1. This result indicates that the interaction frequency of the transgenes is not changed by the presence of T7 RNAP.

To test the efficiency of restriction nuclease digestion, PCR primers BglIIconfw and BglIIconrv were chosen to amplify a 285 bp fragment of genomic DNA containing a *Bgl*II site at its centre. To quantify total DNA, PCR primers rpoZampfw and rpoZamprv were chosen to amplify a 292 bp genomic fragment that did not contain a *Bgl*II site. Restriction digestion efficiency was determined by comparing the ratios of the BglIIconfw/rv fragment∶rpoZampfw/rv fragments in the presence and absence of restriction digestion.

## Supporting Information

Figure S1 **DNA fragments used in ‘pulldown’- and FCS-based assays.** **A.** Diagrams of DNA fragments (i) 800-bp promoter-less control fragment. (ii) 452-bp template. (iii) 290-bp template. (iv) 70-bp template. Numbers indicate the position of elements (in bp) relative to the 5′ ends of the templates. **B.** Transcripts produced by T7 RNAP. The templates in (A) were transcribed in reactions containing 1× KGB, 100 nM template, 200 nM RNAP, and 0.5 mM ATP+GTP+[^32^P]UTP (0.25 µCi/µL) in the presence or absence of 0.5 mM CTP. After 10 min, the resulting RNA was separated by denaturing urea-PAGE, and visualized using a phosphoimager screen (Molecular Dynamics) and a FLA5000 imager (Fuji). (i) Transcripts produced by all three templates. (ii) A second gel better resolving the transcripts produced using the 452-bp template (below). The shorter products produced in reactions lacking CTP indicate that RNAPs transcribe the C-less cassettes but halt at the first C residue.
(TIF)

Figure S2 **The fraction of template occupied by halted RNAPs can be assayed by ‘band shift’.** **A.** A transcription reaction (in buffer LS1) lacking NTPs containing 50 nM T7 RNAP and 8 nM of the 452-bp template (encoding a T7 promoter, a 382-bp C-less cassette, and a C-containing 3′ end) was prepared, and sampled under sequentially-applied conditions. These samples were separated using a native 1.5% agarose gel, and stained with SYBR green I. In the absence of NTPs, the templates are not stably bound by RNAPs, and thus migrate as free DNA (lane 1). Adding ATP+UTP+GTP (to 0.5 mM) causes RNAPs to initiate and halt at the end of the C-less cassette. The templates are now stably bound by RNAPs and their transcripts, and so migrate more slowly (lane 2). Adding CTP (to 0.5 mM) allows RNAPs to ‘run-off’ and vacate most templates, which migrate once again as free DNA (lane 3). DNase treatment shows that RNA makes only a minor contribution to the observed fluorescence (lane 4), while additional RNase treatment removes all nucleic acid (lane 5). **B.** The fraction of template occupied by T7 RNAP in (B) quantified using AIDA image-analysis software (Raytest). For each condition, the amount of occupied template was calculated by subtracting the amount of freely-migrating DNA (as judged by band intensity) from the total amount of DNA (found in lane 1). Repeating the experiment in the buffer KGB instead of LS1 yielded similar results (data not shown).
(TIF)

Figure S3 **RNAPs halt on the 290-bp and 452-bp templates with similar frequencies.** **A.** Transcripts produced during the ‘pulldown’ assay. A transcription reaction (in KGB) containing 0.1 µM biotinylated 452-bp template, 0.1 µM 290-bp template, and 0.3 µM T7 RNAP was initiated by the addition of ATP+GTP+[^32^P]UTP (0.25 µCi/µL) to 0.5 mM in the presence or absence of beads (4.5×10^8^ beads/mL). After 30 s, reactions were halted by the addition of formamide to 80% (v/v), and subjected to denaturing urea-PAGE. Total [^32^P]RNA was then visualized using a phosphoimager screen (Molecular Dynamics) and a FLA5000 imager (Fuji). **B.** Quantitation of the ^32^P incorporated into the transcripts in (A). Initiation rates on the 452-bp and 290-bp templates can be inferred from the intensities of the corresponding transcripts (which measured 382 bp and 243 bp, respectively). When transcript length is accounted for, we see that RNAPs initiated on the 452-bp template at ∼0.7× the rate at which they initiated on 290-bp templates. We conclude that when the majority of 290-bp templates are occupied, a similar fraction of the 452-bp templates will also be occupied.
(TIF)

Figure S4 **The autocorrelation curve of labeled elongation complexes is well fit using a two-dimensional one-species model.** (i) Representative autocorrelation curve (blue, upper panel) recorded using FCS in the experiment of Fig. 2Aiv. A reaction containing 1.75 µM T7 RNAP, 2 nM labeled 70-bp template, and 0.54 µM unlabeled 452-bp template, was initiated by the addition of ATP+UTP+GTP. After RNAPs had halted at the first C residues (30 s), the autocorrelation function of the labeled templates was determined by FCS. (ii) A fit of the autocorrelation function produced in (i) using a two-dimensional one-species model (red, upper panel; equation 1), and yielding a diffusion time of 4.1 ms. Residuals (red, lower panel) are minor, suggesting that the model used to fit the curve is well-suited to the sample (see Materials and methods).
(TIF)

Text S1 **Additional notes and materials and methods.**
(DOC)

Table S1 **Primers used in this study.**
(DOCX)
