# Supplementary figures and images for: T7 RNA Polymerase Functions In Vitro without Clustering
Source: PLoS One. 2012 Jul 2;7(7):e40207. doi: 10.1371/journal.pone.0040207 (PMC3388079; doi:10.1371/journal.pone.0040207)

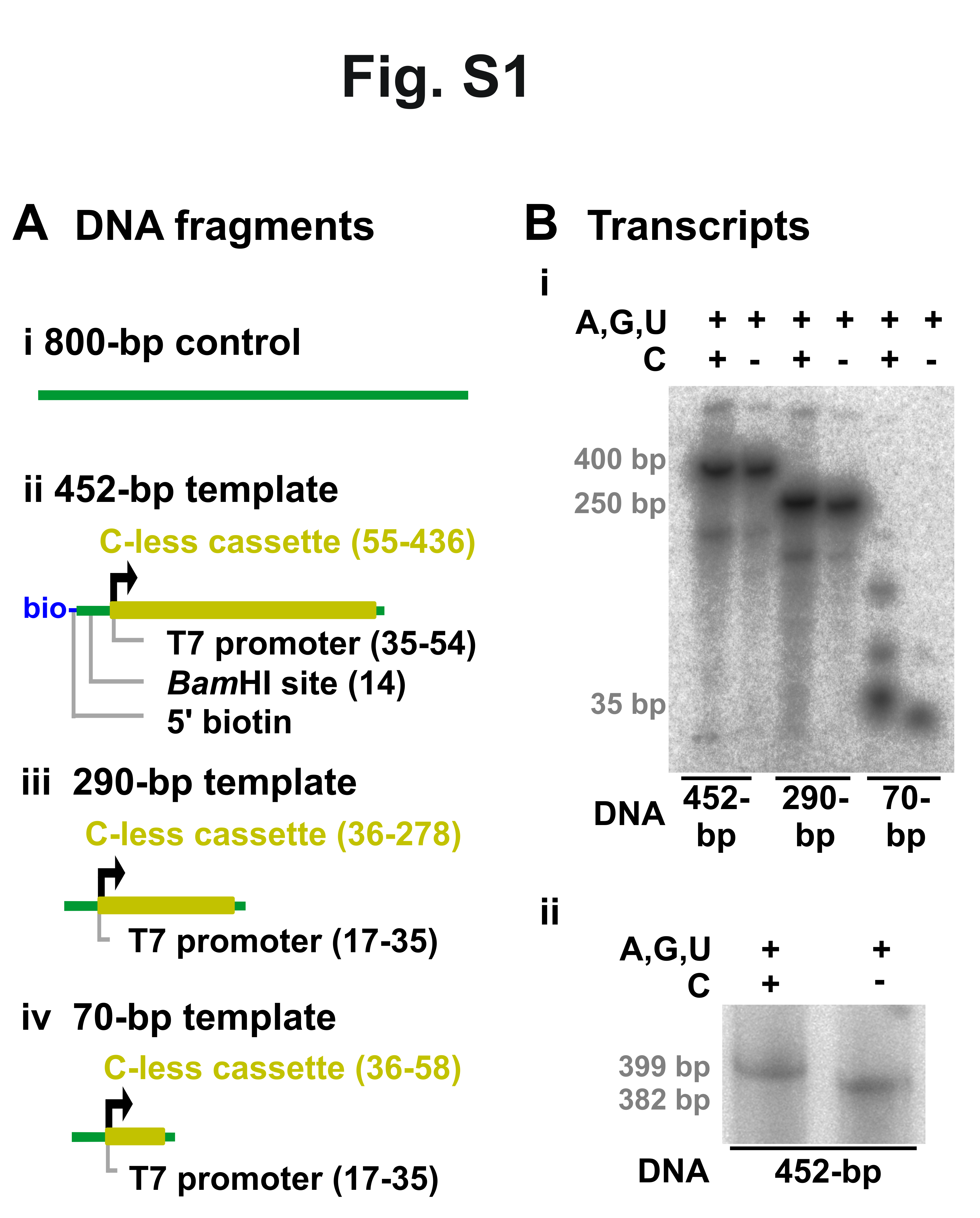

Supplement: Figure S1 — DNA fragments used in ‘pulldown’- and FCS-based assays. A. Diagrams of DNA fragments (i) 800-bp promoter-less control fragment. (ii) 452-bp template. (iii) 290-bp template. (iv) 70-bp template. Numbers indicate the position of elements (in bp) relative to the 5′ ends of the templates. B. Transcripts produced by T7 RNAP. The templates in (A) were transcribed in reactions containing 1× KGB, 100 nM template, 200 nM RNAP, and 0.5 mM ATP+GTP+[32P]UTP (0.25 µCi/µL) in the presence or absence of 0.5 mM CTP. After 10 min, the resulting RNA was separated by denaturing urea-PAGE, and visualized using a phosphoimager screen (Molecular Dynamics) and a FLA5000 imager (Fuji). (i) Transcripts produced by all three templates. (ii) A second gel better resolving the transcripts produced using the 452-bp template (below). The shorter products produced in reactions lacking CTP indicate that RNAPs transcribe the C-less cassettes but halt at the first C residue. (TIF) [file pone.0040207.s001.tif]

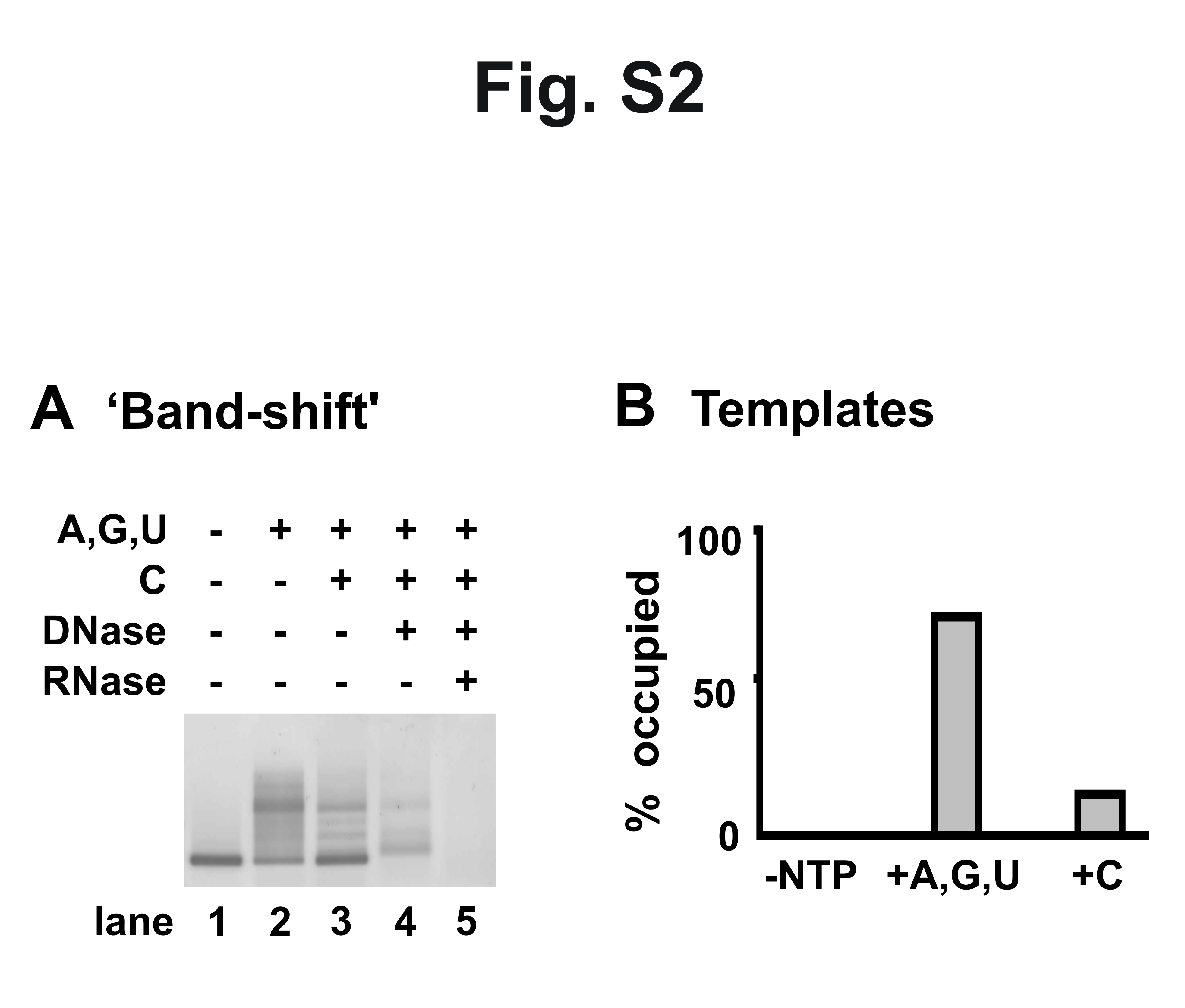

Supplement: Figure S2 — The fraction of template occupied by halted RNAPs can be assayed by ‘band shift’. A. A transcription reaction (in buffer LS1) lacking NTPs containing 50 nM T7 RNAP and 8 nM of the 452-bp template (encoding a T7 promoter, a 382-bp C-less cassette, and a C-containing 3′ end) was prepared, and sampled under sequentially-applied conditions. These samples were separated using a native 1.5% agarose gel, and stained with SYBR green I. In the absence of NTPs, the templates are not stably bound by RNAPs, and thus migrate as free DNA (lane 1). Adding ATP+UTP+GTP (to 0.5 mM) causes RNAPs to initiate and halt at the end of the C-less cassette. The templates are now stably bound by RNAPs and their transcripts, and so migrate more slowly (lane 2). Adding CTP (to 0.5 mM) allows RNAPs to ‘run-off’ and vacate most templates, which migrate once again as free DNA (lane 3). DNase treatment shows that RNA makes only a minor contribution to the observed fluorescence (lane 4), while additional RNase treatment removes all nucleic acid (lane 5). B. The fraction of template occupied by T7 RNAP in (B) quantified using AIDA image-analysis software (Raytest). For each condition, the amount of occupied template was calculated by subtracting the amount of freely-migrating DNA (as judged by band intensity) from the total amount of DNA (found in lane 1). Repeating the experiment in the buffer KGB instead of LS1 yielded similar results (data not shown). (TIF) [file pone.0040207.s002.tif]

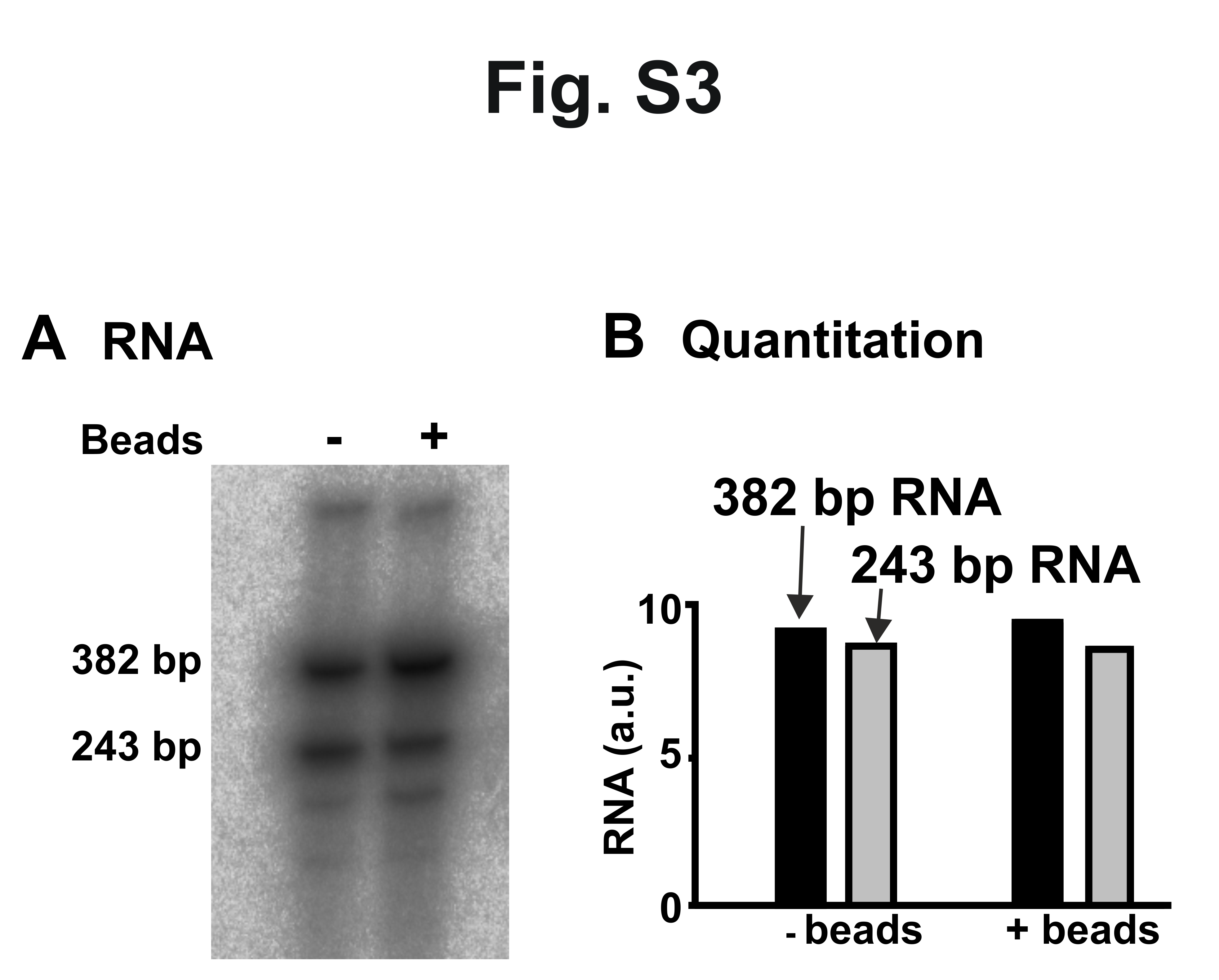

Supplement: Figure S3 — RNAPs halt on the 290-bp and 452-bp templates with similar frequencies. A. Transcripts produced during the ‘pulldown’ assay. A transcription reaction (in KGB) containing 0.1 µM biotinylated 452-bp template, 0.1 µM 290-bp template, and 0.3 µM T7 RNAP was initiated by the addition of ATP+GTP+[32P]UTP (0.25 µCi/µL) to 0.5 mM in the presence or absence of beads (4.5×10^8^ beads/mL). After 30 s, reactions were halted by the addition of formamide to 80% (v/v), and subjected to denaturing urea-PAGE. Total [32P]RNA was then visualized using a phosphoimager screen (Molecular Dynamics) and a FLA5000 imager (Fuji). B. Quantitation of the 32P incorporated into the transcripts in (A). Initiation rates on the 452-bp and 290-bp templates can be inferred from the intensities of the corresponding transcripts (which measured 382 bp and 243 bp, respectively). When transcript length is accounted for, we see that RNAPs initiated on the 452-bp template at ∼0.7× the rate at which they initiated on 290-bp templates. We conclude that when the majority of 290-bp templates are occupied, a similar fraction of the 452-bp templates will also be occupied. (TIF) [file pone.0040207.s003.tif]

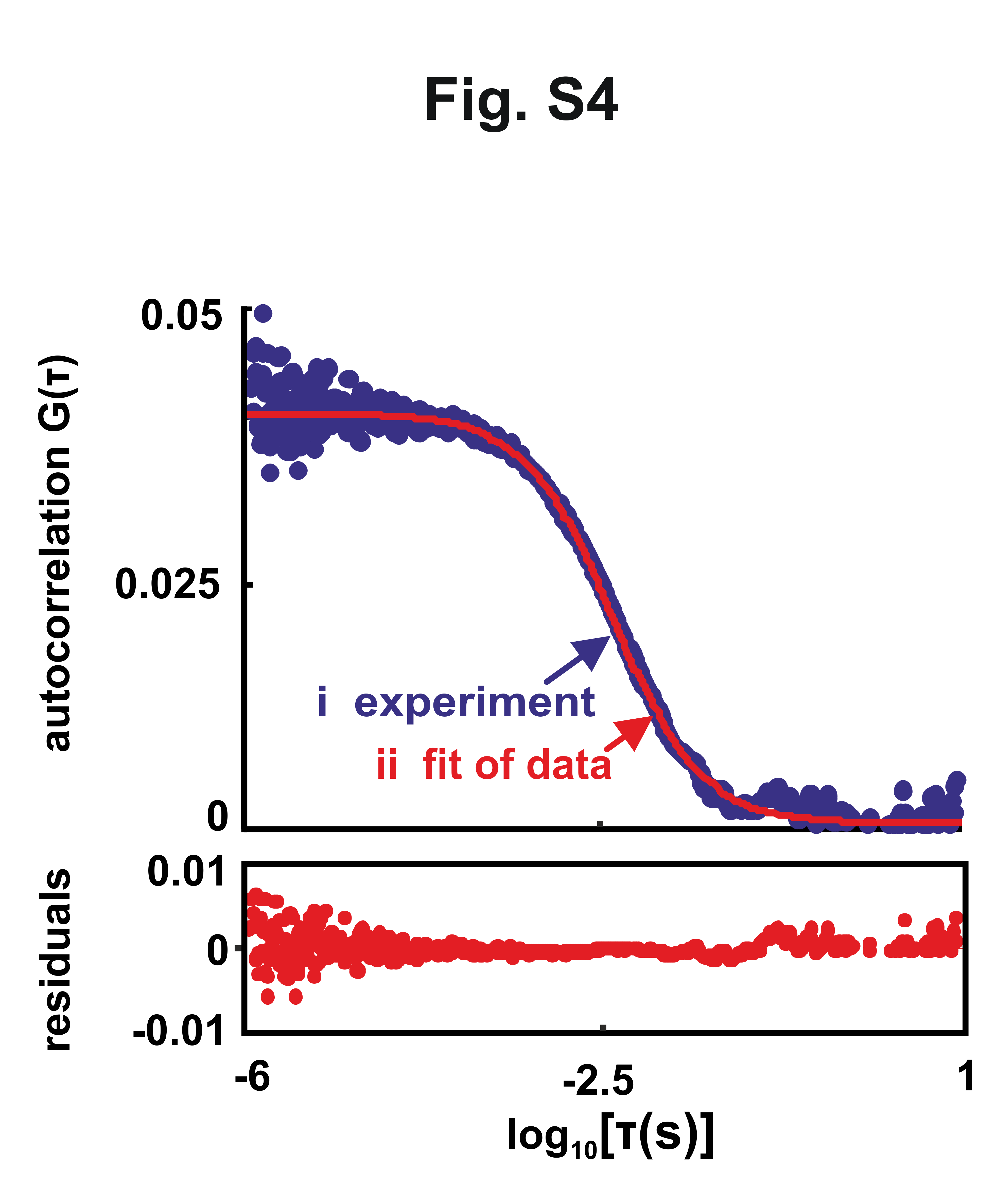

Supplement: Figure S4 — The autocorrelation curve of labeled elongation complexes is well fit using a two-dimensional one-species model. (i) Representative autocorrelation curve (blue, upper panel) recorded using FCS in the experiment of Fig. 2Aiv. A reaction containing 1.75 µM T7 RNAP, 2 nM labeled 70-bp template, and 0.54 µM unlabeled 452-bp template, was initiated by the addition of ATP+UTP+GTP. After RNAPs had halted at the first C residues (30 s), the autocorrelation function of the labeled templates was determined by FCS. (ii) A fit of the autocorrelation function produced in (i) using a two-dimensional one-species model (red, upper panel; equation 1), and yielding a diffusion time of 4.1 ms. Residuals (red, lower panel) are minor, suggesting that the model used to fit the curve is well-suited to the sample (see Materials and methods). (TIF) [file pone.0040207.s004.tif]
