# Supplementary material for: T7 RNA Polymerase Functions In Vitro without Clustering
Source: PLoS One. 2012 Jul 2;7(7):e40207. doi: 10.1371/journal.pone.0040207 (PMC3388079; doi:10.1371/journal.pone.0040207)
Supplement: Text S1 — Additional notes and materials and methods. (DOC) [file pone.0040207.s005.doc]

**Supplementary Text 1**

**Text S1A**

Our observation that the attachment of the 290-bp templates to the beads was disrupted by tRNA suggests that this was not driven by protein-protein interactions between RNAPs. It seems more likely that it was driven by non-specific RNA interactions. We believe that an *in vitro* interaction involving RNA is unlikely to be physiologically relevant, as nascent mRNAs (such as the ones transcribed by T7 RNAP) are covered by ribosomes *in vivo* .

One can imagine several types of artefactual RNA-based interactions that might be responsible for the observed enrichment of the 290-bp template in the pellet. RNA is known to aggregate *in vitro* ; thus the enrichment could easily have been caused by interactions between the nascent transcripts emerging from ECs on the 290-bp and bead-bound 452-bp templates. Unengaged RNAPs also bind directly and non-specifically to RNA ; thus it could be the case that nascent RNA extruded from ECs on 452-bp templates was bound by initiation complexes on 290-bp templates (or *vice versa*). Another possible source of the enrichment was non-specific binding of the 290-bp ECs directly to the beads; we eliminated this possibility by showing that omitting the 452-bp template from the assay also abolished the enrichment (data not shown).

**Text S1B**

The diffusion time of a globular molecule is proportional to its radius ; When two particles bind together, they typically produce a complex with an effective radius smaller than the sum of the radii of the two initial particles. Therefore, the diffusion time of the complex formed by the two particles is likely to be less than the sum of the diffusion times of the individual particles.

**Text S1C**

The diffusion time of the 70-bp template (2.4 ± 0.1 ms) corresponded to a diffusion coefficient of (6.5 ± 1.7)·10^-7^ cm^2^/s (using equation 4). We could not find a measured value for the diffusion coefficient of 70-bp DNA in the literature. However, when, dsDNA is short (i.e., <500 bp), its diffusion time can be accurately calculated (i.e., to within ~10%) by modeling it as a rod; this model predicts diffusion times that are directly proportional to template length . It is thus possible to extrapolate the diffusion time (or diffusion coefficient) of short DNA fragments using an appropriate experimentally-measured standard. We calculated that the expected diffusion coefficient of 70-bp dsDNA was (7 ± 2)·10^-7^ cm^2^/s, by extrapolating from the measured diffusion coefficient of 118 bp DNA (4 ± 1)·10^-7^ cm^2^/s; ). Thus, our experimentally-measured diffusion coefficient was in agreement with previously measured values. The diffusion time of the 452-bp template (15 ms) was similarly extrapolated from the diffusion time of the 70-bp template (2.4 ms). As DNA persistence length (and thus shape) is relatively invariant across different conditions , the linear dependance of DNA diffusion time on DNA length is unlikely to be affected by our choice of buffers.

To estimate the diffusion coefficient of T7 RNAP, we took advantage of the fact that the diffusion behavior of globular proteins is determined mostly by their molecular weight . The *B. subtilis* protein ɑ-amylase (97 kDa) was close to the size of T7 RNAP (99 kDa), and has a known diffusion coefficient of 5·10^-7^ cm^2^/s . We assumed that T7 RNAP would diffuse with a similar speed, and thus would have a diffusion time of 2-3 ms.

**Text S1D**

In our FCS experiment, we induced RNAPs to initiate and halt on C-less cassette-containing templates. The fraction of templates occupied by RNAPs in this experiment can be estimated using only the number of templates and active RNAPs: as halted RNAPs are stable on their templates for >10 min (but take only seconds to initiate; ), we expect that – in the presence of ATP + UTP + GTP – virtually every active RNAP will halt on a template (until every template is full). We note that if one RNAP is ‘bumped’ off its template by a second RNAP , the template will still be occupied by one halted RNAP.

In order to estimate what fraction of our RNAPs were active, we determined their specific activity. Our measured value of 350,000-400,000 U/mg (using a commercial RNAP with known activity as a standard) was close to the specific activity at which every RNAP is active (400,000 U/mg; ). We concluded that >85% of our RNAPs were active. Given this activity, we expect ~85% of templates to be occupied in reactions containing a 1:1 ratio of RNAP:template, and virtually 100% to be occupied in reactions using a 5:1 ratio of RNAP:template.

This expectation was validated when we labeled a cysteine-free mutant of T7 RNAP (with a specific activity of 350,000 U/mg) with Atto647N, and performed a ‘leading edge’ single molecule FRET experiment (as in ) using an excess of the Cy3B-labeled template 70-bp template (data not shown). We found that in the presence of ATP + UTP + CTP, 70% of RNAPs were bound to templates, and produced a high FRET species indicating that they had halted at the first C residue (+23). As expected, addition of CTP abolished this population.

We also note that in Figure 1B, a three-fold excess of RNAP is more than enough to occupy virtually every template in solution. As we used a 5-fold molar excess of RNAP in the FCS-based assay, we are confident that the majority of templates were occupied by halted ECs in these experiments.

**Text S1E**

Although the pBAD-driven T7 RNAP expression system (sold by Invitrogen) is not identical to the IPTG-inducible system used by , manufacturer testing shows that both are equally potent.

**Text S1F**

We imagine several different ways that our 3C-based assay might have missed an interaction between active T7 RNAPs. It is possible that a protein encoded by phage DNA acts as a ‘bridge’ between active RNAPs in order to nuclear EC clusters. Such an interaction would not be detected in our assay, because other proteins encoded by phage DNA were not present. However we do note that yeast-two-hybrid studies found no interaction between the inactive EC and any other phage protein (with the exception of T7 lysozyme, which is a known inhibitor; ).

It is possible that the structure of the *E. coli* chromosome is rigidly defined, and that a T7:T7 interaction was too weak to substantially alter chromatin structure. In eukaryotes, some pairs of active transcription units do not produce a 3C product, probably because they are kept apart by chromosome structure . Indeed, it is known that some parts of the *E. coli* chromosome are unable to come together *in vivo* . However it seems unlikely that this problem would prevent the detection of a strong interaction by our assay. The 3C product detected from the two T7-transcribed genes indicates that – in at least some cells – the two genes are close enough to be cross-linked. Even if the two genes were prevented from coming into contact in – for example, 90% of cells – one would still expect a strong interaction between them to increase the amount of corresponding ligation products that arose from the remaining 10% of cells. However, we also note that an interaction between T7 RNAPs would be missed if it was much weaker than the other forces shaping chromatin structure. In this situation, the overall proximity of the two genes would be unchanged by the interaction.

We are also unable to exclude the possibility that random ‘background’ interactions were prevalent enough to obscure an increase in the crosslinking frequency of the genes. However, as there are ~30 *Bgl*II restriction fragments within 100 kb of the T7 gene 10 integration site, one might expect that a strong interaction of T7 gene 10 with a single fragment (i.e., the fragment containing the YFP gene) would result in an enrichment of the corresponding ligation products.

We were also concerned that low 3C efficiency might affect our results. However we have calculated that our protocol is indeed efficient. 3C in *S. cerevisiae* requires 32 cycles of PCR on 1 µg of template . The 3C protocol employed here uses 35 cycles of PCR – producing roughly 2^3^  = 8 times more product – but uses ~10x less template (30 ng of *E. coli* 3C template contains the same number of genomic copies as 100 ng of yeast template). Thus our 3C is as efficient as the well-optimized protocol used in yeast.

Still, 3C is a complex and poorly-understood technique – even in mammalian and yeast systems, where it is used regularly. Although we have provided arguments against some of the most obvious sources of false negatives, our assay cannot completely eliminate the possibility that active T7 RNAPs cluster *in vivo*.

**Text S1G**

Systems biology approaches have estimated that the concentration of potentially active RNAP (i.e., T7 RNAP not complexed to T7 lysozyme) does not exceed 30 nM during T7 infection . Therefore, our finding that T7 ECs do not interact with a *K_d_* <1 µM suggests that these ECs will also not interact *in vivo*. However it is very difficult to estimate the strength of attraction between ECs that would be necessary to cause clustering *in vivo*. During phage infection, several ECs will be present on the same piece of phage DNA, and so will have an increased effective concentration. However the entropic cost of looping the DNA template would act to prevent clustering, and would need to be overcome by a strong attractive force between RNAPs. ‘Macromolecular crowding’ would also serve to increase the effective concentrations of the ECs . Thus, although the concentration of T7 ECs *in vivo* is known, it is difficult to estimate the strength of interaction that would be necessary to cause clustering.

**Text S1H**

**Supplementary Materials and Methods**

***Preparation of T7 RNA polymerase***

His-tagged T7 RNAP was expressed using the pT7-911 plasmid , and purified using Ni-NTA (Qiagen). Protein was then precipitated by adding (NH_4_)_2_SO_4_ to 25% and spinning at 20,000 * g for 5 min. The pellet was then resuspended in buffer A (40mM KHPO_4_ pH 7.7, 1 mM EDTA, 10 mM DTT) + 600 mM NaCl. Preparations were dialyzed overnight against ‘storage buffer’ (50 mM Tris, pH 8.0, 1 mM EDTA, 5 mM DTT, 0.5 M NaCl, 50% glycerol) and stored at -80°C. Purity was assessed by denaturing gel electrophoresis followed by staining with Coomassie blue, and found to be >90%. Protein concentration was quantified by its absorption at 280 nm using an absorption coefficient of 140,000 M^-1^cm^-1^.

***Quantitative PCR (qPCR)***

qPCR was performed on a Rotor-Gene 3000 thermocycler using Platinum SYBR green I qPCR supermix UDG (Invitrogen). Reactions contained 1x reaction mix, 0.4 µM each primer, and varying amounts of DNA template (≤10 ng). Reactions were heated to (i) 50°C for 3 min, (ii) 95°C for 5 min, (iii) 95°C for 15 s, (iv) 60°C for 15 s, and (v) 60°C for 20 s, while recording SYBR green I fluorescence. Steps (iii)-(v) were then repeated 39 more times. In order to verify the amplification of only the desired amplicon, a melting curve was acquired by raising the temperature from 72°C to 95°C, and the size of PCR products was determined by gel electrophoresis. In all cases, only one product was observed. A standard curve was acquired for each primer by quantitating samples containing known amounts of KF22 genomic DNA (isolated using a Genomic Tip 5000; Qiagen). Comparing the ‘cross-threshold’ (Ct) value of samples to the standard curve allowed absolute quantitation. Standard curves always had ‘R’ (correlation coefficient) values of >0.99. Data was analyzed using Rotorgene 3000.

***Construction of integration cassettes***

T7gene10-CmR was constructed by amplifying T7gene10 from the plasmid pGEMEX-1 (Promega) using primers T7gene10ampprom and T7gene10ampterm, and the cassette encoding chloramphenicol resistance from pROD23 (a gift from Rodrigo Reyes, Oxford) using primers kanamp1fw and kanamp1rv, digesting the fragments separately with *Xma*I, ligating the two together, then gel purifying the desired product. pT7-Ypet-KanR was constructed by amplifying the *Ypet* gene from the plasmid pYpet-His (a gift from Patrick Daugherty, UCSB; ) using primers T7P-Ypetfw and T7P-YpetrvEcoRI, and the kanamycin resistance gene from pROD17 using primers kanamp1fwMfeI and kanamprv1, digesting the products with *EcoR*I and *Mfe*I respectively, then ligating the two together and gel purifying the desired product.

***Genomic manipulation of E. coli***

Insertions into the *E. coli* genome were performed by ‘recombineering’ as described in . Briefly, PCR fragments encoding 50 bp of homology to the genomic sequence upstream of the insertion site, followed by sequence of the element to be inserted, and then followed by 50 bp homologous to the genomic sequence downstream of the insertion site were prepared using Picomaxx DNA polymerase (Stratagene), then purified using Minelute columns (Qiagen) followed by isopropanol precipitation. Competent cells were prepared by growing the strain DY330 to an OD_600_ of 0.4 - 0.6 at 32°C with shaking (200 rpm) followed by 15 min of shaking at 42°C (in a water bath). In a cold room (4°C), cells from 35 mL of culture were pelleted by spinning at 4600 * g for 7 min, washed sequentially with 30 mL and 1 mL of ice-cold 10% glycerol, and resuspended in 200 µL of 10% glycerol. In an ice-cold electroporation cuvette (0.1 cm, Biorad), 50 µL of cells were mixed with roughly 1 µL of 100 ng/µL purified PCR product, and electroporated at 1.8 kV using a Gene Pulser (Biorad). Time constants of successful transformations were always above 5 ms. One millilitre of room temperature LB was immediately added, and the cells were then shaken overnight at 32°C before plating on selective media. Colonies were streaked out, and the insertion loci amplified by colony PCR using primers outside the insertion site. The product was then sequenced to ensure proper insertion and the absence of mutations.

To construct KF1-1, the *galK* locus was replaced with the T7gene10-CmR fragment amplified by the primers T7gene10Cmampfw and T7gene10Cmamprv. To construct strain KF5-1, fragment pT7-Ypet-KanR was amplified using the primers Ypet892700fw and Ypet892700rv, and then inserted into the *E. coli* genome at position 892,700. The ‘3C’ strain KF22-1 was constructed by transducing MG1655 with (i) KF1-1 (chloramphenicol selection), then (ii) KF5-1 (kanamycin selection), then (iii) BL21-AI (tetracycline selection; this strain contained T7 RNAP expressed under an arabinose inducible promoter, and linked to a tetracycline resistance marker; Invitrogen).

***P1 phage transduction***

Transducing loci from one strain to another was performed using P1 phage (a gift from Dave Sheratt, Oxford). To create P1 lysates, 300 µL of saturated overnight culture of the donor strain was mixed with 30 µL 50 mM CaCl_2_ and 100 µL P1 lysate (grown on MG1655), and incubated at 37°C for 20 min. The cells were then added to 5 mL LB containing 5 mM CaCl_2_ and shaken at 37°C. After >6 h, 1 mL of chloroform was added to kill any remaining cells, then removed by spinning at 5000 * g for 10 min and taking the supernatant. Lysates were then stored at 4°C.

Transductions were performed by mixing 900 µL of an overnight culture of the acceptor strain with 100 µL 50 mM CaCl_2_, pelleting the cells at 5000 * g for 1 min, removing 900 µL of the supernatant, then resuspending the pellet and mixing with 50 µL of donor phage lysate. Cells were then incubated for 20 min at 37°C before the addition of 900 µL ‘phage buffer’ (100 mM Na_2_HPO_4_, 22 mM KH_2_PO_4_, 85 mM NaCl, 1 mM MgSO_4_, 0.1 mM CaCl_2_, 0.001% gelatin) at room temperature. Cells were then pelleted at 5000 * g for 1 min, resuspended in LB + 5 mM sodium citrate, shaken for 1-3 h at 30°C or 37°C, plated on LB agar + 5 mM sodium citrate containing the appropriate antibiotics, and then streaked to single colonies twice on the same media to purify away the phage.

***Western blotting***

All primary antibodies were resuspended in 0.5x PBS + 50% glycerol at 1 mg/mL. For detection of T7gp10, membranes were blocked in TBST + 3% BSA, and then incubated with 10 mL 1:10,000 primary antibody (mouse monoclonal; Novagen 69522) in TBST. For detection of the RNA polymerase of bacteriophage T7, membranes were blocked in TBST + 5% low fat milk, and then incubated with 20 mL 1:3,000 primary antibody (mouse monoclonal; Novagen 69522) in TBST. Addition of 20 µg of anti-NusA antibody (mouse monoclonal; Neoclone W0010) along with primary antibodies allowed the detected protein to be used as a loading control.

**References in Text S1**

1. French SL, Miller OL, Jr. (1989) Transcription mapping of the Escherichia coli chromosome by electron microscopy. Journal of Bacteriology 171: 4207-4216.

2. Szewczak AA, White SA, Gewirth DT, Moore PB (1990) On the use of T7 RNA polymerase transcripts for physical investigation. Nucleic Acids Research 18: 4139-4142.

3. He B, Rong M, Durbin RK, McAllister WT (1997) A mutant T7 RNA polymerase that is defective in RNA binding and blocked in the early stages of transcription. Journal of Molecular Biology 265: 275-288.

4. Lakowicz JR (2006) Principles of fluorescence spectroscopy. New York: Springer. xxvi, 954 p. p.

5. Bjorling S, Kinjo M, Foldes-Papp Z, Hagman E, Thyberg P, et al. (1998) Fluorescence correlation spectroscopy of enzymatic DNA polymerization. Biochemistry 37: 12971-12978.

6. Stellwagen NC, Magnusdottir S, Gelfi C, Righetti PG (2001) Measuring the translational diffusion coefficients of small DNA molecules by capillary electrophoresis. Biopolymers 58: 390-397.

7. Lu Y, Weers B, Stellwagen NC (2002) DNA persistence length revisited. Biopolymers 61: 261-275.

8. Krouglova T, Vercammen J, Engelborghs Y (2004) Correct diffusion coefficients of proteins in fluorescence correlation spectroscopy. application to tubulin oligomers induced by Mg2+ and paclitaxel. Biophysical Journal 87: 2635-2646.

9. Young ME, Carroad PA, Bell RL (1980) Estimation of diffusion coefficients of proteins. Biotechnology and Bioengineering 22: 947-955.

10. Mentesana PE, Chin-Bow ST, Sousa R, McAllister WT (2000) Characterization of halted T7 RNA polymerase elongation complexes reveals multiple factors that contribute to stability. Journal of Molecular Biology 302: 1049.

11. Zhou Y, Martin CT (2006) Observed instability of T7 RNA polymerase elongation complexes can be dominated by collision-induced "bumping". Journal of Biological Chemistry 281: 24441-24448.

12. Martin CT, Coleman JE (1987) Kinetic-analysis of T7 RNA-polymerase promoter interactions with small synthetic promoters. Biochemistry 26: 2690-2696.

13. Kapanidis AN, Margeat E, Laurence TA, Doose S, Ho SO, et al. (2005) Retention of transcription initiation factor s70 in transcription elongation: single-molecule analysis. Molecular Cell 20: 347-356.

14. Studier FW, Moffatt BA (1986) Use of bacteriophage T7 RNA polymerase to direct selective high-level expression of cloned genes. Journal of Molecular Biology 189: 113-130.

15. Bartel PL, Roecklein JA, SenGupta D, Fields S (1996) A protein linkage map of Escherichia coli bacteriophage T7. Nature Genetics 12: 72-77.

16. Papantonis A, Larkin JD, Wada Y, Ohta Y, Ihara S, et al. (2010) Active RNA polymerases: mobile or immobile molecular machines? PLoS Biology 8: e1000419.

17. Valens M, Penaud S, Rossignol M, Cornet F, Boccard F (2004) Macrodomain organization of the Escherichia coli chromosome. EMBO Journal 23: 4330-4341.

18. Miele A, Gheldof N, Tabuchi TM, Dostie J, Dekker J (2006) Mapping chromatin interactions by chromosome conformation capture. Current Protocols in Molecular Biology Chapter 21: 1-11.

19. Endy D, Kong D, Yin J (1997) Intracellular kinetics of a growing virus: a genetically structured simulation for bacteriophage T7. Biotechnology and bioengineering 55: 375-389.

20. Zhou HX, Rivas GN, Minton AP (2008) Macromolecular crowding and confinement: biochemical, biophysical, and potential physiological consequences. Annual Review of Biophysics 37: 375-397.

21. Abramochkin G, Shrader TE (1995) The leucyl/phenylalanyl-tRNA-protein transferase. Journal of Biological Chemistry 270: 20621-20628.

22. He B, Rong M, Lyakhov D, Gartenstein H, Diaz G, et al. (1997) Rapid mutagenesis and purification of phage RNA polymerases. Protein Expression and Purification 9: 142-151.

23. Nguyen AW, Daugherty PS (2005) Evolutionary optimization of fluorescent proteins for intracellular FRET. Nature Biotechnology 23: 355-360.

24. Sharan SK, Thomason LC, Kuznetsov SG, Court DL (2009) Recombineering: a homologous recombination-based method of genetic engineering. Nature Protocols 4: 206-223.
