# Supplementary material for: T7 RNA Polymerase Functions In Vitro without Clustering
Source: PLoS One. 2012 Jul 2;7(7):e40207. doi: 10.1371/journal.pone.0040207 (PMC3388079; doi:10.1371/journal.pone.0040207)
Supplement: Table S1 — Primers used in this study. (DOCX) [file pone.0040207.s006.docx]

**Table S1**

| Primer name | Sequence (5’-3’) |
| --- | --- |
| KRF36 | GATTACGCCAAGCTCTAATACGACTCACTATAGGGAGAGGTGAGGAGAGGATAAGG |
| KRF37 | GATCTGACTGGGATTTCCCAAATCTATCATCATTC |
| KRF3 | GATTACGCCAAGCTCTAATACGACTCACTATAGGGA GAGGGAGTGGAATGAGAAATGAG |
| KRF28 | TCCTCGTTGGTGGTGTGTCTCCATACCCTTCCTCCATC |
| KRF32 | [biotin]CGCAGAACTGCTCGGATCCGATTACGCCAAGCTC |
| KRF47 | ACGCCAAGCTCTAATACGACTCACTATAGGGAGAGGGAGAGGTAAGTTGAGAAATGAGCACACCACCAAC |
| KRF42 | ACGCCAAGCTCTAATACGA |
| KRF43 | GTTGGTGGTGTGCTCATTTC |
| KRF45 | GT[amine dT]GGTGGTGTGCTCATTTC |
| T7gene10CmRampfw | GTTTGCGCGCAGTCAGCGATATCCATTTTCGCGAATCCGGAGTGTAAGAACGACGGCCAGTGCCAAGC |
| T7gene10CmRamprv | CGGCTGACCATCGGGTGCCAGTGCGGGAGTTTCGTTCAGCACTGTCCTGCAGATCTCGATCCCGCGAAATTAATACGAC |
| Ypet892700fw | TGCAAAATTATCTGCTGTTTTTAACCTTTTCTTAAAGATTATTTCACTTCGATGTCGCTAATACGACTCACTATAGG |
| Ypet892700rv | AAATTTTTATGCATATTTGCTCTATGTGATAAAGCCAAATCGACACAAGAAAGCTTGCATGCCTGCAGGTCGACTCTAGAGG |
| T7gene10ampprom | CAATACGCAAACCGCCTCTCC |
| T7gene10ampterm | ATCTAGCCCGGGTTGCCGATTTCGGCCTATTGG |
| T7P-Ypetfw | GATGTCGCTAATACGACTCACTATAGGGAGAGGGAGTGAGGAGTGAAACGATGTCTAAAGG |
| T7P-Ypetrv | GCGTACATGACGAATTCAAAAAACCCCTCAAGACCCGTTTAGAGGCCCCAAGGGGGGCCACCTTGGCCTTAGTGG |
| KF101to8BglIIfw | CCCAACCGGATGCCATATCGAC |
| KF101to8BglIIrv | GTACTGCGCGACTGCGCTTAA |
| KF101to16BglIIfw | AGCTTGCAGTGGGCTTAC |
| KF101to16BglIIrv | CATTGCAGCGGCATTTGG |
| 3CposA | TGGCCCGATACAATTTACCG |
| 3CposB | GAAAGATCAGGGCATTGACC |
| BglIIconfw | ACGTTGGATCCCAAGACAAG |
| BglIIconrv | CGTTGCCAATGGATATGGTCTG |
| rpoZampfw | GCGCGAAATCGAAGAAGGTC |
| rpoZamprv | TACCGGGTGCGTGATATAGG |
